# Involvement of mast cells in monocrotaline-induced pulmonary hypertension in rats

**DOI:** 10.1186/1465-9921-12-60

**Published:** 2011-05-02

**Authors:** Bhola K Dahal, Djuro Kosanovic, Christina Kaulen, Teodora Cornitescu, Rajkumar Savai, Julia Hoffmann, Irwin Reiss, Hossein A Ghofrani, Norbert Weissmann, Wolfgang M Kuebler, Werner Seeger, Friedrich Grimminger, Ralph T Schermuly

**Affiliations:** 1 University of Giessen Lung Centre (UGLC), Giessen, Germany; 2 Institute of Physiology, Charité-Universitaetsmedizin Berlin, Germany; 3 Department of Pediatric Surgical Intensive Care, Erasmus MC-Sophia Children's Hospital, Rotterdam, Netherlands; 4 The Keenan Research Centre at the Li Ka Shing Knowledge Institute of St. Michael's Hospital, Toronto, Canada; 5 Max-Planck-Institute for Heart and Lung Research, Bad Nauheim, Germany

## Abstract

**Background:**

Mast cells (MCs) are implicated in inflammation and tissue remodeling. Accumulation of lung MCs is described in pulmonary hypertension (PH); however, whether MC degranulation and c-kit, a tyrosine kinase receptor critically involved in MC biology, contribute to the pathogenesis and progression of PH has not been fully explored.

**Methods:**

Pulmonary MCs of idiopathic pulmonary arterial hypertension (IPAH) patients and monocrotaline-injected rats (MCT-rats) were examined by histochemistry and morphometry. Effects of the specific c-kit inhibitor PLX and MC stabilizer cromolyn sodium salt (CSS) were investigated in MCT-rats both by the preventive and therapeutic approaches. Hemodynamic and right ventricular hypertrophy measurements, pulmonary vascular morphometry and analysis of pulmonary MC localization/counts/activation were performed in animal model studies.

**Results:**

There was a prevalence of pulmonary MCs in IPAH patients and MCT-rats as compared to the donors and healthy rats, respectively. Notably, the perivascular MCs were increased and a majority of them were degranulated in lungs of IPAH patients and MCT-rats (p < 0.05 versus donor and control, respectively). In MCT-rats, the pharmacological inhibitions of MC degranulation and c-kit with CSS and PLX, respectively by a preventive approach (treatment from day 1 to 21 of MCT-injection) significantly attenuated right ventricular systolic pressure (RVSP) and right ventricular hypertrophy (RVH). Moreover, vascular remodeling, as evident from the significantly decreased muscularization and medial wall thickness of distal pulmonary vessels, was improved. However, treatments with CSS and PLX by a therapeutic approach (from day 21 to 35 of MCT-injection) neither improved hemodynamics and RVH nor vascular remodeling.

**Conclusions:**

The accumulation and activation of perivascular MCs in the lungs are the histopathological features present in clinical (IPAH patients) and experimental (MCT-rats) PH. Moreover, the accumulation and activation of MCs in the lungs contribute to the development of PH in MCT-rats. Our findings reveal an important pathophysiological insight into the role of MCs in the pathogenesis of PH in MCT- rats.

## Background

A growing body of studies in recent years implicates inflammation and dysregulated growth factor signaling in the pathogenesis of pulmonary arterial hypertension (PAH) [1]. Among the growth factors, platelet derived growth factor (PDGF) has been extensively investigated [2,3]. We have demonstrated that reversal of experimental pulmonary hypertension (PH) and vascular remodeling by imatinib is associated with the inhibition of PDGF receptor (PDGFR), a member of receptor tyrosine kinase (RTK) family [3]. Subsequently, Wang *et al. *have found that c-kit play an important role in systemic vascular remodeling [4,5]. As imatinib is also a potent inhibitor of the RTK, c-kit [6], the data indicate that c-kit may potentially contribute to the pathological remodeling of pulmonary vessels.

It is well documented that hematopoetic stem and progenitor cells express c-kit; however, c-kit expression is downregulated on maturation of all haemopoietic lineages, except mast cells (MCs) that retain high levels of expression [7]. In general, c-kit activation initiates cellular responses such as chemotaxis, proliferation, differentiation and survival [8]. Moreover, activation of c-kit by its ligand, the stem cell factor (SCF)/MC growth factor is associated with MC development, proliferation, migration and degranulation [9,10]. Therefore, c-kit is described as a pharmacological target for therapy of multiple pathological conditions linked to MCs[11]. MC activation and degranulation have been attributed a role in airway and cardiac remodeling [12-15]. Regarding pulmonary vascular pathology, an increased lung MCs has been reported in plexogenic pulmonary arteriopathy [16], pulmonary hypertension [17] and congenital heart diseases associated with early pulmonary vascular diseases [18]. Moreover, MCs/c-kit expressing cells have been localized along the periphery/adventitial layer of remodelled pulmonary vessels in experimental PH [19-21]. Recently, MC degranulation has been implicated both in the development of pulmonary vascular remodeling in chronic hypoxic rats and in the regression of the remodeling upon bringing them back to normoxia [22,23]. Activated MCs produce several mediators including the biogenic amine serotonin, the cytokines interleukin (IL)-6 and IL-13, and the serine proteases chymase and tryptase that are capable of activating matrix metalloproteases (MMPs) [9]. The implication of serotonin, IL-6, IL-13 and MMPs in PH pathogenesis [1,24-26] provides a potential mechanistic rationale to the hypothesis that MCs may be involved in the pathogenesis of PH and pulmonary vascular remodeling. However, a systematic examination of pulmonary MCs in clinical and experimental PH and an elucidation of the role of MCs in animal model of progressive PH are still missing. In this study, we therefore investigated the lung tissues from idiopathic PAH (IPAH) patients and monocrotaline (MCT)-injected rats to determine total and perivascular MC count, and the degranulation of the perivascular MCs. Furthermore, we investigated the effects of the pharmacologic inhibitions of c-kit and MC degranulation on hemodynamics, right ventricular hypertrophy and pulmonary vascular remodeling in MCT-induced PH in rat.

## Methods

### Animals and experimental design

Adult male Sprague-Dawley (SD) rats were obtained from Charles River Laboratories, Germany. All studies were approved by the local authority (Regierungspräsidium Gießen) and were performed according to the guidelines of the University of Giessen. PH was induced in rats by MCT injection as described [3]. Rats were randomized and treated daily with the selective c-kit inhibitor (PLX, kindly provided by Plexxikon Inc.). PLX was freshly prepared in 5% DMSO, 1% methylcellulose and administered by oral gavage at the dose of 50 mg/kg body weight. Another group of rats received the MC stabilizer Cromolyn sodium salt (CSS, Sigma-Aldrich) daily through intra-peritoneal injection. CSS was freshly prepared in saline and given at the dose of 40 mg/kg body weight. Rats in the placebo groups received respective vehicles only. In a preventive approach, pharmacological inhibition of c-kit or MC degranulation was initiated from day 1 till day 21 of MCT injection. In the therapeutic approach, the inhibition was performed from day 21 till day 35 of MCT injection when the disease is established or already rapidly progressing.

### Hemodynamic and Right Ventricular Hypertrophy (RVH) Measurements

Hemodynamic and RVH measurements were performed as previously reported [27]. For monitoring hemodynamics, rats were anesthetized, tracheotomized and artificially ventilated at a constant frequency of 60 breaths per minute. Inspiratory oxygen (FIO2) was set at 0.5, and a positive end expiratory pressure of 1.0 cm H_2_O was used. The left carotid artery was isolated and cannulated with a polyethylene cannula connected to a fluid-filled force transducer and the systemic arterial pressure (SAP) was measured. A catheter was inserted through the right jugular vein into the right ventricle to measure right ventricular systolic pressure (RVSP). The animals were ex-sanguinated and the lungs were flushed with sterile saline to get rid of blood. The left lung was fixed for histology in 3.5% neutral buffered formalin and the right lung was snap frozen in liquid nitrogen. The heart was isolated and dissected under microscope. The right ventricular wall was separated from the left ventricular wall and ventricular septum. Dry weight of the right ventricle, free left ventricular wall and ventricular septum was determined. Right ventricular hypertrophy was expressed as the ratio of weight of the right ventricular wall (RV) and that of the free left ventricular wall and ventricular septum (LV+S).

### Histology and Pulmonary Vascular Morphometry

Lung histology and vascular morphometry were performed as described [27]. The formalin-fixed and paraffin-embedded lung tissues were subject to sectioning to yield 3 μm thick sections. Elastica staining was performed according to common histopathological procedures. The degree of muscularization of small peripheral pulmonary arteries was assessed by double-staining the sections with an anti-α-smooth muscle actin antibody (dilution 1:900, clone 1A4, Sigma, Saint Louis, Missouri) and anti-human von Willebrand factor antibody (vWF, dilution 1:900, Dako, Germany) followed by analysis of the vessels using a computerized morphometric analysis system (QWin; Leica, Germany) to determine the degree of pulmonary artery muscularization. In each rat, 80 to 100 intra-acinar arteries (20 to 50 μm diameter) were categorized as muscular, partially muscular, or non-muscular. In addition, arteries of the same size were analyzed to determine the medial wall thickness as previously described [27]. All analyses were done in a blinded fashion.

### Patient characterization

Human lung tissues were obtained from donors and patients with IPAH undergoing lung transplantation. After explantation, lung tissues were formalin-fixed and paraffin-embedded according to common tissue processing protocol. Written informed consent was obtained from each individual patient or the patient's next kin. Among the IPAH patients six were male and four were female. They had mean pulmonary artery pressure (mPAP in mmHg) of 74.10 ± 9.8 (mean ± SEM, n = 10) and cardiac index (CI in l/min/m^2^) of 2.09 ± 0.22 (mean ± SEM, n = 8). The patients had undergone treatment for PAH with the currently available options namely, prostacyclin analogues, PDE5 inhibitor and endothelin receptor antagonists. The study protocol was approved by the ethics committee of the University of Giessen that conforms to the principles outlined in the Declaration of Helsinki.

### Histology and mast cell (MC) counting

In addition to the paraffin-embedded lung tissues from IPAH patients and donors, the lung tissues of rats from the interventional studies were included in the histology and subsequent MC analysis. Moreover, lung tissues from MCT-rats that received imatinib (100 mg/kg bw/day through oral gavage) by a therapeutic approach were included. To identify MCs toluidine blue staining was performed using standard protocols. Briefly, paraffin-embedded tissue sections were dewaxed, rehydrated and incubated with 0.05% w/v toluidine blue for 2-3 minutes. MC density was quantified by counting the number of toluidine blue-positive MCs. MC numbers and the extent of their degranulation were assessed manually as described in the literature [28,29] with modification. Total MCs were counted throughout section in each lung under light microscope. In addition, perivascular MCs (of different vessel sizes such as 20-50, 50-150 and >150 μm) were counted. Moreover, perivascular MCs were categorized into granulated and degranulated based on the extrusion of secretory granules (i.e. intact MCs with dense cytoplasm are granulated, whereas degranulated MCs have light cytoplasm with empty spots due to the discharge of secretory granules). Furthermore, an index of granulation (IOG) [(number of granulated MC/number of degranulated MC)] was determined. The IOG was expressed in percentage assuming that the average IOG in donors and healthy rats were 100%. The MC analysis was done by independent investigators. The methods and results of the MC analysis have been presented in the form of an abstract [30].

### Data analysis

Data were expressed as mean ± SEM. Comparison among the experimental groups were done by one way analysis of variance (ANOVA) and subsequent Newman-Keuls test. Unpaired T-test was used to compare MC count. A value of P < 0.05 was considered as statistically significant. The number of animals/tissue samples used in each experiment/analysis has been mentioned in the figure legends.

## Results

### Prevalence and degranulation of MCs in the lungs of IPAH patients

**Figure 1:**
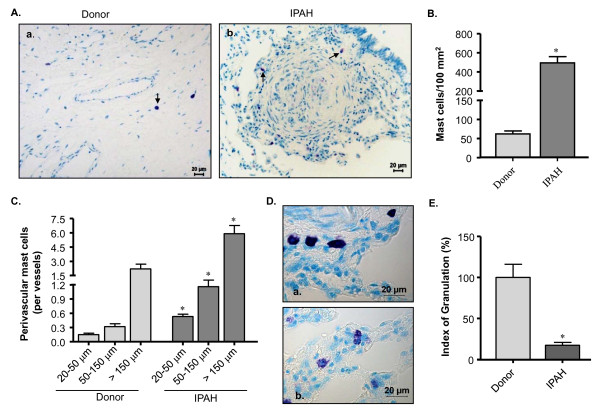
**Prevalence of pulmonary MCs in IPAH patients**. Lung tissues from donors and IPAH patients were stained with toluidine blue (TB). The arrow indicates the positive signal (purple/violet stain) for the TB-stained MCs. (A) Representative photomicrographs of lung sections from donor (a) and patients (b) are shown. (B) Total and (C) perivascular MC count of different vessel size are given. (D) Perivascular MCs were further analyzed to identify granulated (a) and degranulated (b) MCs, and an index of granulation (IOG) was determined. (E) IOG (in %) is shown. Each bar represents Mean ± SEM (n = 10-15). *p < 0.05 versus donor/corresponding vessels of donor. Scale = 20 μm.

Toluidine blue staining showed that MCs were scattered throughout the lung tissues including peribronchial, septal and perivascular areas (Figure 1A). We counted the MCs and found that MC population was about 8 fold higher in IPAH patients as compared to the donors (Figure 1B). There was a preponderance of perivascular MCs in IPAH lungs (p < 0.05 versus donor lungs). Moreover, about 3 and 4 fold increases in MCs were observed around resistance (20-50 and 50-150 μm in diameter) and the larger (> 150 μm) vessels, respectively in the lungs of IPAH patients as compared to the donors (Figure 1C). We categorized perivascular MCs as granulated and degranulated (Figure 1D) and calculated the index of granulation (IOG) to examine their activation status. Interestingly, there was a 5.7 fold decrease of IOG in IPAH lungs (Figure 1E), suggesting that majority of the perivascular MCs were degranulated/activated.

### Prevalence and degranulation of MCs in the lungs of MCT-rats

**Figure 2:**
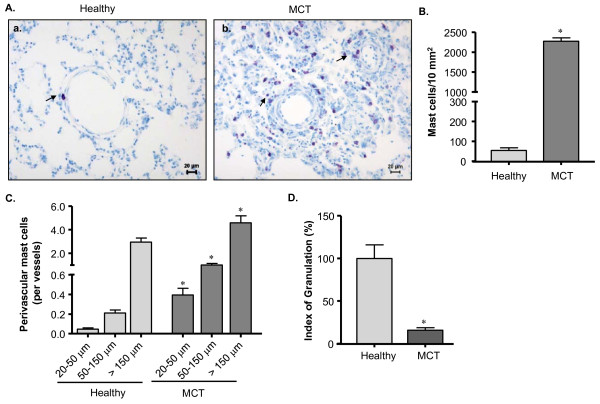
**Prevalence of pulmonary MCs in MCT-rats**. Lung tissue from healthy and MCT-rats (that received single injection of saline and monocrotaline, respectively) were stained with toluidine blue (TB). The arrow indicates the positive signal (purple/violet stain) for MCs. (A) Representative photomicrographs of healthy (a) and MCT-injected (b) rat lungs are shown. (B) Total and (C) perivascular MC count of different vessel sizes are given. An IOG was determined for perivascular MCs and (D) IOG (in %) is shown. Each bar represents Mean ± SEM (n = 10). *p < 0.05 versus healthy rats/corresponding vessels of healthy rats. Scale = 20 μm.

As in clinical PH, pulmonary MC count was increased in MCT-rats (p < 0.05 versus healthy rats) and they were distributed throughout the lungs including peribronchial, perivascular and septal areas (Figure 2A, 2B). Perivascular MCs, the MC population of interest, was prevalent in MCT-rats (p < 0.05 versus healthy rats). Interestingly, there was about 9-fold increase in the number of MCs around the intra-acinar vessels (20-50 μm in diameter), whereas about 5- and 2-fold increases were found around the pre-acinar vessels (50-150 and >150 μm, respectively) in MCT-rats as compared to healthy rats (Figure 2C). As observed in the IPAH lungs, majority of the perivascular MCs was activated as evident from 6.3 fold decrease of their IOG in MCT-rats as compared to healthy rats (Figure 2D).

### Effects of inhibition of c-kit and MC degranulation in MCT-rats: Preventive approach

#### Hemodynamics, right ventricular hypertrophy and pulmonary vascular remodeling

**Figure 3:**
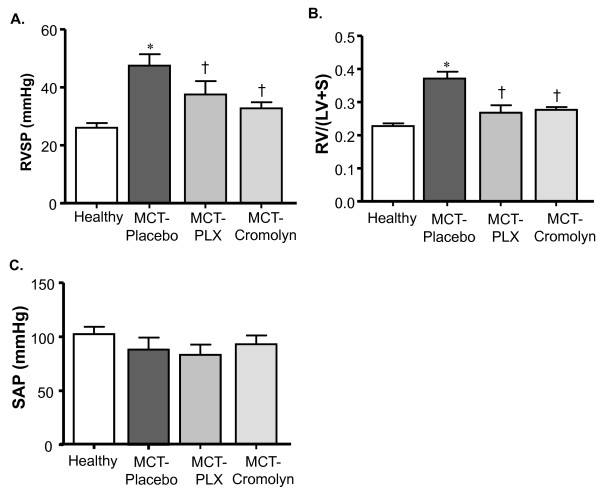
**Effects of inhibiting c-kit and MC degranulation on PH and right ventricular hypertrophy (RVH) of MCT-rats**. Rats were treated with selective c-kit inhibitor (PLX), MC stabilizer (Cromolyn) or placebo from day 1 to 21 after MCT-injection. The rats in healthy group received saline injection instead of MCT. (A) Right ventricular systolic pressure (RVSP), (B) Right to left ventricular plus septum weight ratio (RV/(LV+S)) and (C) Systemic arterial pressure (SAP)are given. Each bar represents Mean ± SEM (n = 8-10). *p < 0.05 versus healthy; ^†^p < 0.05 versus MCT-placebo.

We then investigated if inhibition of c-kit by PLX and of MC degranulation by CSS had any modulating effects on the development of MCT-induced PH and vascular remodeling. We found that MCT-rats receiving placebo developed significantly higher right ventricular systolic pressure (RVSP) and right ventricular hypertrophy (RV/(LV+S)) as compared to healthy rats, whereas rats treated with PLX and CSS revealed significantly reduced RVSP and RV/(LV+S) as compared to placebo group (Figure 3A, 3B). No significant change was observed in systemic arterial pressure (SAP) (Figure 3C).

**Figure 4:**
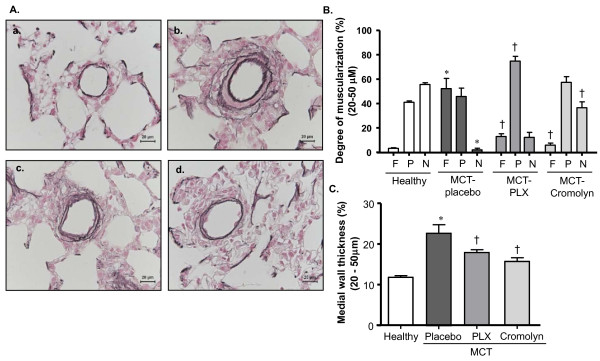
**Effects of inhibiting c-kit and MC degranulation on pulmonary vascular remodeling of MCT-rats**. Rats were treated with selective c-kit inhibitor (PLX), MC stabilizer (Cromolyn) or placebo from day 1 to 21 after MCT-injection. The rats in healthy group received saline injection instead of MCT. Double immunostaining for von Willebrand factor and α-smooth muscle actin, and elastica staining were performed on the lung tissues followed by vascular morphometry. (A) Representative photomicrographs of elastica-stained lung tissues (healthy- a, placebo- b, PLX- c and Cromolyn- d) are shown. (B) Proportion of non- (N), partially (P) or fully (F) muscularized pulmonary arteries and their (C) medial wall thicknesses (%) are given. Each bar represents Mean ± SEM (n = 8-10). *p < 0.05 versus healthy; ^†^p < 0.05 versus MCT-placebo. Scale bar = 20 μm.

An increased muscularization and medial wall thickness of distal pulmonary vessels was present in MCT-rats receiving placebo as reflected from the enhanced immunoreactivity for α-smooth muscle cell (SMC) actin (not shown) and elastica staining (Figure 4A). Vascular morphometry revealed an increased fully muscularized vessels accompanied by decreased non-muscularized vessels in placebo group (P < 0.05 versus healthy rats). In rats receiving PLX and CSS, the percentage of fully muscularized vessels was reduced (P < 0.05 versus placebo) (Figure 4B). Moreover, the medial wall thickness was increased in the placebo group (p < 0.05 versus healthy rats). Corroborating the decreased fully muscularized vessels, medial wall thickness was significantly reduced in rats receiving PLX and CSS (Figure 4C).

#### MC count and degranulation

**Figure 5:**
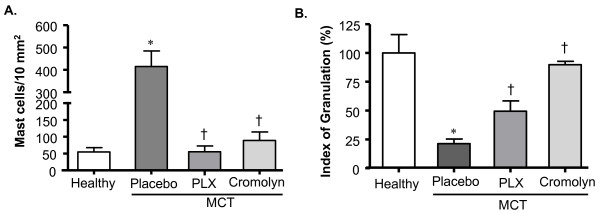
**Effects of MC stabilizer and c-kit inhibitor on pulmonary mast cells in rats with MCT- induced PH**. Rats were treated with selective c-kit inhibitor (PLX), MC stabilizer (Cromolyn) or placebo from day 1 to 21 after MCT-injection. The rats in healthy group received saline injection instead of MCT. The lung tissue sections were stained with toluidine blue (TB). The TB-stained MCs were counted throughout the tissue sections and (A) total MCs were determined. Perivascular MCs were examined to determine the index of granulation (IOG). (B) Index of granulation (in %) is given. Each bar represents Mean ± SEM (n = 6-8). *p < 0.05 versus healthy; ^†^p < 0.05 versus MCT-placebo.

We investigated the effects of treatments on pulmonary MCs. The number of MCs in MCT-rats receiving placebo was increased as compared with the healthy rats, whereas there was a decrease of MCs in MCT-rats treated with PLX and CSS (p < 0.05 versus placebo) (Figure 5A). The perivascular MCs were then analyzed and their activation/degranulation status was determined. We found that the IOG of perivascular MCs was significantly decreased in placebo rats as compared to healthy rats. Treatment with PLX and CSS resulted in an increase in IOG (p < 0.05 versus placebo) (Figure 5B).

### Effects of inhibition of c-kit and MC degranulation in MCT-rats: Therapeutic approach

#### Hemodynamics, right ventricular hypertrophy and pulmonary vascular remodeling

**Figure 6:**
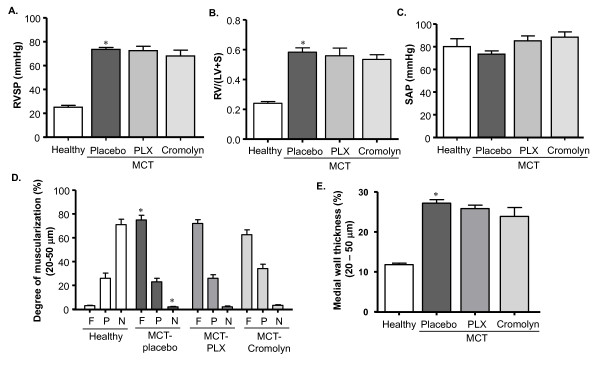
**Effects of inhibiting c-kit and MC degranulation on PH, right ventricular hypertrophy (RVH) and pulmonary vascular remodeling of MCT-rats**. Rats were treated with selective c-kit inhibitor (PLX), mast cell stabilizer (Cromolyn) or placebo from day 21 to 35 after MCT-injection followed by hemodynamic and RVH measurement. The rats in healthy group received saline injection instead of MCT. (A) Right ventricular systolic pressure (RVSP), (B) right to left ventricular plus septum weight ratio (RV/(LV+S)) and (C) systemic arterial pressure (SAP) are shown. Double immunostaining for von Willebrand factor and α-smooth muscle actin, and elastica staining were performed on the lung tissues followed by vascular morphometry. (D) Proportion of non- (N), partially (P) or fully (F) muscularized pulmonary arteries and their (E) medial wall thicknesses (%) are given. Each bar represents Mean ± SEM (n = 8-10). *p < 0.05 versus healthy; ^†^p < 0.05 versus MCT-placebo group.

The findings of the preventive study prompted us to investigate the effects of inhibition of c-kit and MC degranulation by a therapeutic approach. Surprisingly, we did not find any significant reduction of RVSP and RV/(LV+S) in MCT-rats treated with PLX and CSS as compared to placebo rats (Figure 6A, 6B and 6C). Moreover, the treatment did not impair the progression of pulmonary vascular remodeling as evident from the comparable degree of muscularization and medial wall thickness of distal pulmonary vessels in treated versus placebo rats (Figure 6D and 6E).

#### MC count and degranulation

**Figure 7:**
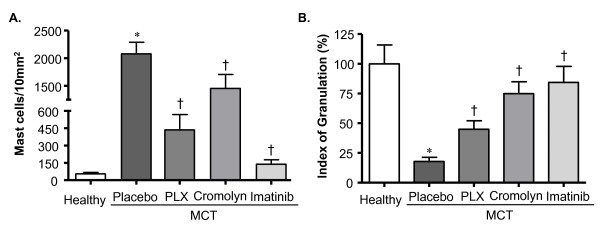
**Effects of inhibiting c-kit and MC degranulation on pulmonary MCs in rats with MCT- induced PH**. Rats were treated with selective c-kit inhibitor (PLX), MC stabilizer (Cromolyn), imatinib or placebo from day 21 to 35 after MCT-injection. The rats in healthy group received saline injection instead of MCT. The lung tissue sections were stained with toluidine blue (TB). The TB-stained MCs were counted throughout the tissue sections and (A) total MCs were determined. Perivascular MCs were examined to determine the index of granulation (IOG). (B) IOG (in %) is given. Each bar represents Mean ± SEM (n = 6-8). *p < 0.05 versus healthy; ^†^p < 0.05 versus MCT-placebo group. Scale bar = 20 μm.

We analyzed pulmonary MCs including lung tissues obtained from imatinib-treated MCT-rats. A massive increase of MCs was found in MCT-rats receiving placebo, whereas treatments with PLX, CSS and imatinib significantly reduced MC counts (Figure 7A). Analysis of perivascular MCs revealed that the IOG was significantly decreased in placebo rats (P < 0.05 versus healthy rats) and the treatments with PLX, CSS and imatinib significantly increased the IOG as compared to placebo (Figure 7B).

## Discussion

For more than a decade, pulmonary MCs are known to accumulate in primary plexogenic pulmonary arteriopathy (PPA) [16], pulmonary hypertension [17] and congenital heart diseases associated with early pulmonary vascular diseases [18]; however, quantitative data on pulmonary MCs have been lacking in the PH patients. In line with the literature, we found higher MC count, suggesting that MCs were prevalent in the lungs of IPAH patients. Additionally, analysis of the perivascular MCs revealed that MC count was significantly higher and majority of them were degranulated in patients. As in IPAH patients, increased pulmonary MCs were observed in MCT-rats. The MC count was significantly higher along the perivascular space and in particular, a remarkable increase was observed around intra-acinar vessels. Corroborating our findings, Miyata *et al. *have described more MCs around the vessels in MCT-rats [31]. Moreover, MCs are localized around the pulmonary vessels in rats with severe PH [21]. We extended these findings and demonstrated that majority of the perivascular MCs were degranulated in the lungs of MCT-rats. The preponderance of the degranulated MCs may be attributable to the potent toxic effects of monocrotaline on the pulmonary vessels resulting in radical tissue injury and inflammatory process [32,33] and increased pulmonary vascular pressure/resistance [34]. Taken together, the findings suggest that a prevalence of degranulated perivascular MCs is common to clinical and experimental PH.

Activation of the receptor c-kit is involved in MC development, proliferation, migration and degranulation, and several pathological conditions related to MC disorders are associated with c-kit dysregulation [9,35-37]. We therefore targeted c-kit and found that the selective inhibition of c-kit by a preventive approach improved PH, RVH and pulmonary vascular remodeling in MCT-rats; furthermore, there was significant reduction in MC accumulation and perivascular MC degranulation in the lungs. The findings suggest that c-kit is involved in the development of PH in MCT-rats by promoting perivascular MC accumulation and degranulation in the lungs. In agreement with our data, Wang *et al. *demonstrate that early intervention with imatinib, a tyrosine kinase inhibitor that beside other RTKs also targets c-kit results in a marked reduction in intimal hyperplasia [4]. Moreover, imatinib treatment from later phase of hyperplasia does not yield beneficial effects [4]. Consistent with the data of Wang *et al*., we observed no beneficial effects of c-kit inhibition by a therapeutic approach in MCT-rats. On the other hand, multikinase inhibitors such as imatinib and sorafenib provide therapeutic benefit in experimental PH [3,38], suggesting that inhibitions of other RTKs like PDGFR or Raf may be attributable to the observed benefits. Indeed, we have previously demonstrated that imatinib provides therapeutic benefit in experimental PH through an inhibition of PDGFR activation [3]. However, whether imatinib has any effects on pulmonary MCs has yet been undetermined. We therefore analyzed lung tissues from MCT-rats treated with imatinib and found that MC accumulation and perivascular MC degranulation were almost abrogated. The effects of imatinib on pulmonary MCs may be attributable to the inhibition of c-kit and to an interference of the interaction of MCs with other factors [39-42]. It is not unlikely that the therapeutic benefits of imatinib in experimental PH may be attributed to its potent effects on MCs, in addition to its effects on vascular cells through inhibition of PDGF signaling. However, we currently do not have evidence to delineate the therapeutic benefits associated with the effects of imatinib on pulmonary MCs.

As c-kit is also expressed by hematopoetic stem/progenitor cells, the effects of its inhibition may not necessarily be due to an interference with MCs [43]. On the other hand, MC activation and degranulation release various mediators including serotonin, cytokines (e.g., IL-6, IL-13), serine proteases (e.g., chymase) and matrix metalloproteases (e.g., MMP 13) [9,44]. Notably, these mediators play a role in the pathogenesis of PH and pulmonary vascular remodeling [1,17,24,25,44]. We therefore selectively inhibited MC degranulation and found that the development of PH, RVH and pulmonary vascular remodeling in MCT-rats was significantly impaired. There was a significant inhibition of perivascular MC degranulation and reduction of pulmonary MC count, suggesting that the amelioration of PH may be associated with the reduced accumulation of MCs and prevention of their mediators from being released. Moreover, the findings suggest that the MC activation and accumulation are mutually enhanced in the process of PH pathogenesis. Corroborating our findings, Hoffmann et al. showed that prevention of MC degranulation attenuated the PH and vascular remodeling in rats with left heart disease and in MCT-rats [34]. The study, however, did not investigate the effects by a therapeutic strategy. In the current study, we inhibited the MC degranulation by a therapeutic approach, but did not observe beneficial effects, suggesting that MCs may no longer have modulating effects on the pathogenesis after the PH is established in MCT-rats. Although surprising at first glance, it should be noted that the intervention was started after the PH was established. The pathogenesis at this advanced stage may be potentially self-perpetuating owing to the involvement of a host of redundant factors. Such factors include various growth factors, proteases and inflammatory mediators that have been incriminated in the pathogenesis of PH [1,3,21,45]. In line with our findings, Banasova et al. demonstrate that MCs play a role in the development of PH in hypoxic rats [22]. The authors observe that inhibition of MC degranulation at an early stage of hypoxia attenuates the development of PH while it is without effects if administered at a later stage. In contrast, Mungal did not observe a beneficial effect on the right ventricular hypertrophy in chronic hypoxic rats by using disodium cromoglycate (DSCG) [46]. This contrasting observation may be attributable to the lower dose of DSCG (10 mg/kg BW) used in his study. Taken together, it may be deduced that an inhibition of MC degranulation impairs the development but does not affect the established PH in rats irrespective of the stimuli (hypoxia/monocrotaline). Our pharmacological inhibition studies (c-kit and MC degranulation) are consistent and thus substantiate the findings that an interference with MC dysfunctions impairs the development of MCT-induced PH. Moreover, our findings reveal a hitherto unrecognized role of MCs in the early development versus late established stages of pathogenesis of MCT-induced PH in rats.

We performed additional studies on mice that were genetically deficient in MCs [47] (c-kit deficient W/W^v ^and stem cell factor deficient Sl/Sl^d ^mice). W/W^v ^mice have a primary defect in hematopoietic stem cells, whereas Sl/Sl^d ^mice have defective tissue microenvironment [47,48]. We exposed the mice to chronic hypoxia and found that both W/W^v ^and Sl/Sl^d ^mice developed PH, RVH and vascular remodeling (Additional file 1*Methods and Results; *Additional file 2, *Figure S1; *Additional file 3, *Figure S2*). Our findings are in line with previous findings from mouse model of hypoxic PH [49] but not from rat models of PH [22,34]. The contrasting findings may be attributable to the relative paucity of MCs in the normal mouse than rats. Indeed, a wide variability in the pulmonary MC numbers has been reported among various species [50]. This raises the issue of the relevance of animal models to clinical situation in humans. The chronic hypoxic mice develop mild PH and vascular remodeling as compared to the MCT-rats, which show several features of human PH such as inflammation, media hypertrophy, adventitial thickening, and progressive increase in pulmonary arterial pressure and right heart failure. We now report that the accumulation and increased degranulation of perivascular MCs in the lungs are common to MCT-rats and IPAH patients. The limitation of our findings is that targeting c-kit and MC degranulation do not provide therapeutic benefits. However, future in *vitro *and *in vivo *studies designed to elucidate MC-associated pathomechanism in pulmonary vascular remodeling at the level of cellular interaction and intracellular signaling may unravel novel potential targets for PH treatment.

In conclusion, the accumulation and activation of perivascular MCs are the histopathological features present in the lungs of IPAH patients and MCT-rats. This, to our knowledge, is the first study that reports the quantitative assessment of pulmonary MCs in clinical and experimental PH. Moreover, the accumulation and activation of MCs in the lungs contribute to the development of PH in MCT-rats. This study offers important pathophysiological insights into the role of MCs in the pathogenesis of PH in MCT- rats.

## Competing interests

The authors declare that they have no competing interests.

## Authors' contributions

BKD, HAG, NW, WS, FG and RTS conceived and designed the study. BKD, DK, CK, and TC performed experiments. BKD, DK, RS, HAG, NW, WS, FG and RTS analyzed and interpreted data. JH, IR and WMK, were involved in interpretation of data. BKD and RTS drafted and finalized the manuscript. DK, RS, HAG, NW, WS, FG, JH, IR and WMK were involved in revising the manuscript for important intellectual content. All authors read and approved the final manuscript.

## Supplementary Material

Additional file 1 **Methods, results and figure legends**. Effects of mast cell (MC) deficiency on chronic hypoxia-induced PH in mice. Click here for file

Additional file 2 **Figure S1**. Effects of c-kit/MC deficiency on chronic hypoxia-induced PH. Click here for file

Additional file 3 **Figure S2**. Effects of stem cell factor/MC deficiency on chronic hypoxia-induced PH. Click here for file
