# Supplementary material for: Involvement of mast cells in monocrotaline-induced pulmonary hypertension in rats
Source: Respir Res. 2011 May 2;12(1):60. doi: 10.1186/1465-9921-12-60 (PMC3104382; doi:10.1186/1465-9921-12-60)
Supplement: Additional file 1 — Methods, results and figure legends. Effects of mast cell (MC) deficiency on chronic hypoxia-induced PH in mice. [file 1465-9921-12-60-S1.DOC]

**Involvement of mast cells in monocrotaline-induced pulmonary hypertension in rats**

Bhola K Dahal^1^, Djuro Kosanovic^1^, Christina Kaulen^1^, Teodora Cornitescu^1^, Rajkumar Savai^1^, Julia Hoffmann^2^, Irwin Reiss^3^, Hossein A Ghofrani^1^, Norbert Weissmann^1^, Wolfgang M. Kuebler^2,4^, Werner Seeger^1,5^, Friedrich Grimminger^1^ and Ralph T Schermuly^1,5^

^1^University of Giessen Lung Centre (UGLC), Giessen, Germany

^2^Institute of Physiology, Charité-Universitaetsmedizin Berlin, Germany

^3^Department of Pediatric Surgical Intensive Care, Erasmus MC-Sophia Children’s Hospital, Rotterdam, Netherlands

^4^The Keenan Research Centre at the Li Ka Shing Knowledge Institute of St. Michael´s Hospital, Toronto, Canada

^5^Max-Planck-Institute for Heart and Lung Research, Bad Nauheim, Germany

**Additional Online Data**

**Methods**

The animal model study were approved by the local authority (Regierungspräsidium Gießen) and were performed according to the guidelines of the University of Giessen.

**Experimental design**

Eight week old wild type (C57BL/6J), C-kit deficient (WBB6F1-W/W^v^), stem cell factor deficient (WBB6F1-Sl/Sl^d^) and their colony control (WBB6F1-^+/+^) mice (Jackson Laboratory). PH was induced in mice by exposing them to normobaric hypoxia (10% inspired O_2_ fraction) while control mice were kept under normoxia (21% inspired O_2_ fraction) in a ventilated chamber for 4 weeks as described previously (1). The level of hypoxia was held constant by an auto regulatory control unit (model 4010, O2 controller, (Labotect; Göttingen, Germany) supplying either nitrogen or oxygen. Excess humidity in the recirculating system was prevented by condensation in a cooling system. CO2 was continuously removed by soda lime. Cages were opened once a day for cleaning as well as for food and water supply.

**Hemodynamic and right ventricular hypertrophy measurements**

Hemodynamic and RVH measurements were performed as previously reported (2). For monitoring hemodynamics, mice were anesthetized, tracheotomized and artificially ventilated. The left carotid artery was isolated and cannulated with a polyethylene cannula connected to a fluid-filled force transducer and the systemic arterial pressure (SAP) was measured. A catheter was inserted through the right jugular vein into the right ventricle for measurement of right ventricular systolic pressure (RVSP). The animals were ex-sanguinated and the lungs were flushed with sterile saline to get rid of blood. The left lung was fixed for histology in 3.5 % neutral buffered formalin and the right lung was snap frozen in liquid nitrogen. The heart was isolated and dissected under microscope. The right ventricular wall was separated from the left ventricular wall and ventricular septum. Dry weight of the right ventricle, free left ventricular wall and ventricular septum was determined. Right ventricular hypertrophy was expressed as the ratio of weight of the right ventricular wall (RV) and that of the free left ventricular wall and ventricular septum (LV+S).

**Histology and pulmonary vascular morphometry**

Lung histology and vascular morphometry were performed as described (2). The formalin-fixed lungs were subject to paraffin embedding. The paraffin-embedded tissues were subject to sectioning to yield 3 µm thick sections. Elastica staining was performed according to common histopathological procedures. The degree of muscularization of small peripheral pulmonary arteries was assessed by double-staining the 3 μm sections with an anti-α-smooth muscle actin antibody (dilution 1:900, clone 1A4, Sigma) and antihuman von Willebrand factor antibody (vWF, dilution 1:900, Dako, Germany) followed by analysis of the vessels using a computerized morphometric analysis system (QWin; Leica, Germany) to determine the degree of pulmonary artery muscularization. 80-100 intra-acinar vessels at a size between 20 and 70 µm accompanying either alveolar ducts or alveoli were analyzed. Each vessel was categorized as muscular, partially muscular, or non-muscular. Additionally, arteries of the same size in the elastic stained lung tissue sections were analyzed to calculate the medial wall thickness (2). All analyses were done in a blinded fashion.

**results**

**Effects of c-kit/mast cell deficiency on chronic hypoxia-induced PH in mice**

We employed the mouse model of chronic hypoxia-induced PH and investigated wild type (WT) and genetically c-kit deficient (W/W^v^) mice, which are also deficient in MCs. Exposure to chronic hypoxia increased RVSP in WT and W/W^v^ mice significantly as compared to their normoxic control (Figure S1 A). There was no significant change in SAP of WT and W/W^v^ mice under normoxia and hypoxia (Figure S1 B). The increased RVSP was accompanied by RVH as evident from significantly increased RV/(LV+S) in hypoxic WT and W/W^v^ mice as compared to the normoxic control (Figure S1 C). Vascular morphometry showed an increased fully muscularized vessels and a decreased the non-muscularized vessels in hypoxic WT and W/W^v^ mice (p<0.05 versus normoxic control) (Figure S1 D). In agreement with the muscularization, there was a significant increase in the medial wall thickness of pulmonary vessels in hypoxic WT and W/W^v^ mice as compared to normoxic controls (Figure S1 E). The W/W^v^ mice were anemic as reflected by the low hematocrit; however, there was a similar response of WT and W/W^v^ mice to hypoxia with about 1.6 and 1.4 fold increases in hematocrit, respectively (Figure S1 F).

**Effects of stem cell factor/mast cell deficiency on chronic hypoxia-induced PH in mice**

We also investigated mice deficient in stem cell factor (SCF), the ligand for c-kit. The SCF deficient mice (Sl/Sl^d^) are genetically deficient in mast cells. Chronic hypoxic exposure resulted in significantly increased RVSP in WT and Sl/Sl^d^ mice as compared to their normoxic control (Figure S2 A). The SAP of WT and Sl/Sl^d^ mice under normoxia and under hypoxia did not show any significant difference (Figure S2 B). There was a significantly increased RV/(LV+S) ratio in hypoxic WT and Sl/Sl^d^ mice as compared to the normoxic mice (Figure S2 C). Pulmonary vascular morphometry revealed that fully muscularized vessels were significantly increased accompanied by a decrease in non-muscularized vessels in hypoxic WT and Sl/Sl^d^ mice (p<0.05 versus normoxic control) (Figure S2 D). Corroborating the muscularization, there was a significant increase in medial wall thickness of pulmonary vessels in hypoxic WT and Sl/Sl^d^ mice as compared to their normoxic controls (Figure S2 E). The Sl/Sl^d^ mice were anemic; however, both Sl/Sl^d^ and WT mice revealed a similar response to the hypoxia with 1.4 fold higher hematocrit values than the normoxic control (Figure S2 F).

**REFERENCES**

(1) Schermuly RT, Dony E, Ghofrani HA, Pullamsetti S, Savai R, Roth M, Sydykov A, Lai YJ, Weissmann N, Seeger W, Grimminger F. **Reversal of experimental pulmonary hypertension by PDGF inhibition.** J Clin Invest 2005,**115**:2811-21.

(2) Dahal BK, Cornitescu T, Tretyn A, Pullamsetti SS, Kosanovic D, Dumitrascu R, Ghofrani HA, Weissmann N, Voswinckel R, Banat GA, Seeger W, Grimminger F, Schermuly RT. **Role of epidermal growth factor inhibition in experimental pulmonary hypertension.** Am J Respir Crit Care Med 2010,**181**(2):158-67.

(3) Chen R, Ning G, Zhao ML, Fleming MG, Diaz LA, Werb Z, Liu Z. **Mast cells play a key role in neutrophil recruitment in experimental bullous pemphigoid.** J Clin Invest 2001,**108**:1151-8.

(4) Bot I, de Jager SC, Zernecke A, Lindstedt KA, van Berkel TJ, Weber C, Biessen EA. **Perivascular mast cells promote atherogenesis and induce plaque destabilization in apolipoprotein E-deficient mice.** Circulation 2007,**115**:2516-25.

**FIGURE LEGENDS**

**Figure S1.** **Effects of c-kit/MC deficiency on chronic hypoxia-induced PH, right ventricular hypertrophy and vascular remodeling.** c-kit deficient (W/W^v^) and their wild type (WT) control mice were exposed to hypoxia for 4 weeks or remained in normoxia throughout (normoxic control) followed by hemodynamic and RV/ (LV+S) measurements. (A) RVSP, (B) SAP and (C) RV/ (LV+S) ratio are shown. Elastica staining and double immuostaining for von Willebrand factor and α-smooth muscle actin were performed in the lung tissues of the mice followed by vascular morphometry. (D) Proportion of non- (N), partially (P) or fully (F) muscularized pulmonary arteries and (E) their medial wall thicknesses (%) are given. (F) The hematocrit values of the mice are shown. Each bar represents Mean ± SEM (n = 8-10). *p<0.05 versus corresponding normoxic control. Nox-normoxia, Hox-hypoxia.

**Figure S2.** **Effects of stem cell factor/MC deficiency on chronic hypoxia-induced PH, right ventricular hypertrophy and pulmonary vascular remodeling.** Stem cell factor deficient (Sl/Sl^d^) and their colony control wild type (WT) mice were exposed to hypoxia for 4 weeks or remained in normoxia throughout (normoxic control) followed by hemodynamic and RV/ (LV+S) measurements. (A) RVSP, (B) SAP and (C) RV/ (LV+S) ratio are shown. Elastica staining and double immuostaining for von Willebrand factor and α-smooth muscle actin were performed in the lung tissues of the mice followed by vascular morphometry. (D) Proportion of non- (N), partially (P) or fully (F) muscularized pulmonary arteries and (E) their medial wall thicknesses (%) are given. (F) The hematocrit values of the mice are shown. Each bar represents mean ± SEM (n= 6-9). *P< 0.05 vs. corresponding normoxic control; ^†^P<0.05 vs. corresponding genotype control. Nox-normoxia, Hox-hypoxia
