# Supplementary material for: Involvement of mast cells in monocrotaline-induced pulmonary hypertension in rats
Source: Respir Res. 2011 May 2;12(1):60. doi: 10.1186/1465-9921-12-60 (PMC3104382; doi:10.1186/1465-9921-12-60)
Supplement: Additional file 2 — Figure S1. Effects of c-kit/MC deficiency on chronic hypoxia-induced PH. [file 1465-9921-12-60-S2.PPT]

## Slide 1
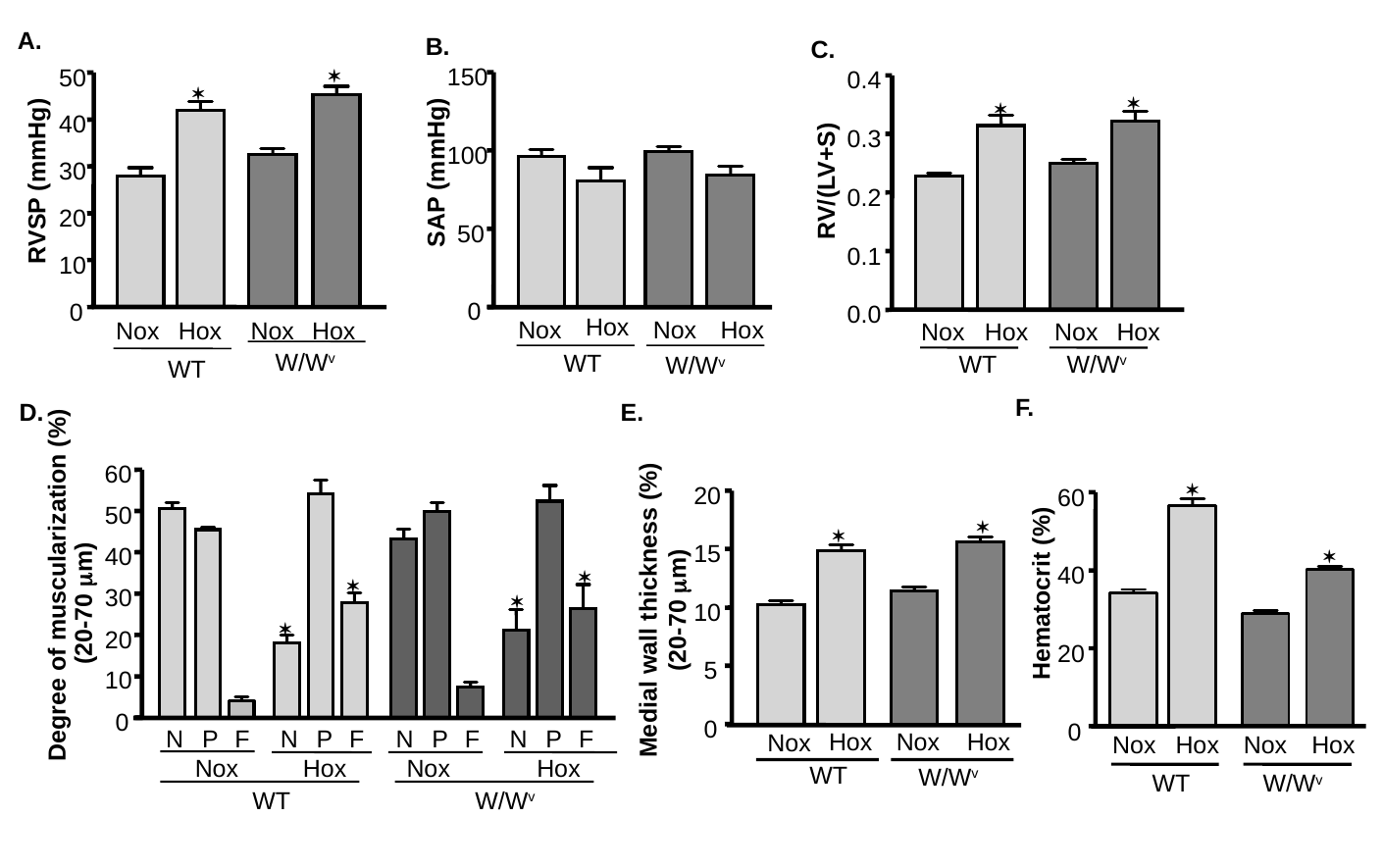

A.
B.
C.

50

40
30
RVSP (mmHg)
20
10
0
Nox
Hox
Nox
Hox
W/Wv
WT
150
100
SAP (mmHg)
50
0
Hox
Nox
Nox
Hox
WT
W/Wv
0.4


0.3
RV/(LV+S)
0.2
0.1
0.0
Nox
Hox
Nox
Hox
WT
W/Wv
F.
E.
D.
Degree of muscularization (%)
(20-70 μm)
60
50
40
30
20
10
0




N
P
F
N
P
F
N
P
F
N
P
F
Nox
Nox
Hox
Hox
W/Wv
WT
20


15
Medial wall thickness (%)
(20-70 μm)
10
5
0
Nox
Hox
Hox
Nox
WT
W/Wv

60

40
Hematocrit (%)
20
0
Nox
Hox
Nox
Hox
WT
W/Wv
