# Supplementary material for: Involvement of mast cells in monocrotaline-induced pulmonary hypertension in rats
Source: Respir Res. 2011 May 2;12(1):60. doi: 10.1186/1465-9921-12-60 (PMC3104382; doi:10.1186/1465-9921-12-60)
Supplement: Additional file 3 — Figure S2. Effects of stem cell factor/MC deficiency on chronic hypoxia-induced PH. [file 1465-9921-12-60-S3.PPT]

## Slide 1
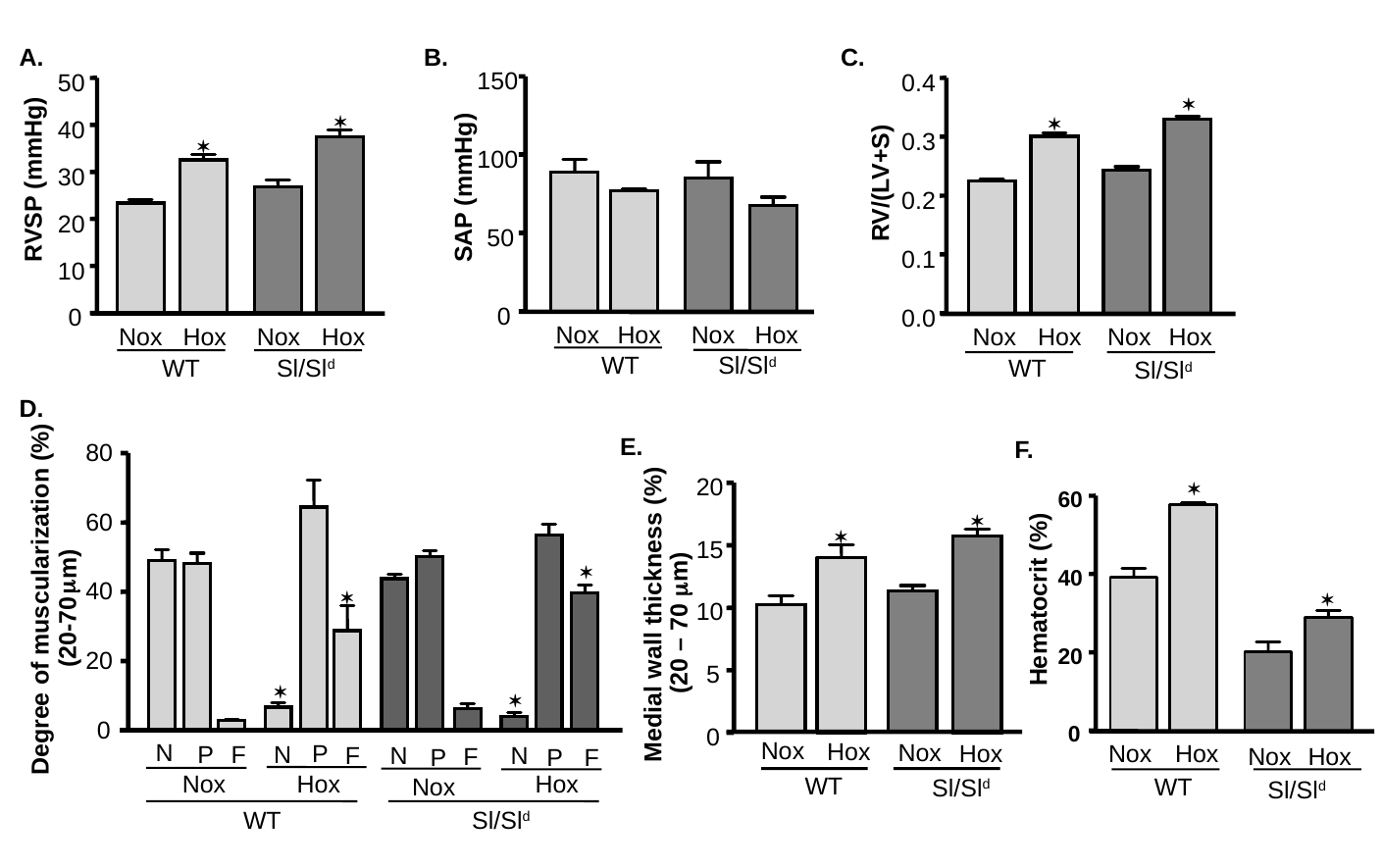

C.
A.
B.
150
100
SAP (mmHg)
50
0
Nox
Hox
Nox
Hox
WT
Sl/Sld
50

40

30
RVSP (mmHg)
20
10
0
Nox
Hox
Nox
Hox
WT
Sl/Sld
0.4


0.3
RV/(LV+S)
0.2
0.1
0.0
Nox
Hox
Nox
Hox
WT
Sl/Sld
D.
E.
m)

Degree of muscularization (%)
(20-70
80
P
60
P

F
N
P
P

F
N
40
20

N

N
F
0
F
Nox
Hox
Hox
Nox
Sl/Sld
WT
F.

60
40

Hematocrit (%)
20
0
Nox
Hox
Nox
Hox
WT
Sl/Sld
Medial wall thickness (%)
(20 – 70 μm)
20
15
10
5
0


Nox
Hox
Nox
Hox
WT
Sl/Sld
